# Unveiling the Potential of Marine Biopolymers: Sources, Classification, and Diverse Food Applications

**DOI:** 10.3390/ma16134840

**Published:** 2023-07-05

**Authors:** Ipsheta Bose, Nousheen, Swarup Roy, Pallvi Yaduvanshi, Somesh Sharma, Vinay Chandel, Deblina Biswas

**Affiliations:** 1 School of Bioengineering and Food Sciences, Shoolini University, Solan 173229, India; ipsheta18@gmail.com (I.B.); nousheengreenstone@gmail.com (N.); yaduvanship123@gmail.com (P.Y.); someshsharma@shooliniuniversity.com (S.S.); vinay85chandel@gmail.com (V.C.); 2 Department of Food Technology and Nutrition, School of Agriculture, Lovely Professional University, Phagwara 144411, India; 3 Department of Instrumentation and Control Engineering, Dr. B. R. Ambedkar National Institute of Technology Jalandhar, Jalandhar 144011, India; deblinabi@gmail.com

**Keywords:** marine biopolymer, biodegradable materials, seaweeds, protein and polysaccharide, food applications

## Abstract

Environmental concerns regarding the usage of nonrenewable materials are driving up the demand for biodegradable marine biopolymers. Marine biopolymers are gaining increasing attention as sustainable alternatives in various industries, including the food sector. This review article aims to provide a comprehensive overview of marine biopolymers and their applications in the food industry. Marine sources are given attention as innovative resources for the production of sea-originated biopolymers, such as agar, alginate, chitin/chitosan, and carrageenan, which are safe, biodegradable, and are widely employed in a broad spectrum of industrial uses. This article begins by discussing the diverse source materials of marine biopolymers, which encompass biopolymers derived from seaweed and marine animals. It explores the unique characteristics and properties of these biopolymers, highlighting their potential for food applications. Furthermore, this review presents a classification of marine biopolymers, categorizing them based on their chemical composition and structural properties. This classification provides a framework for understanding the versatility and functionality of different marine biopolymers in food systems. This article also delves into the various food applications of marine biopolymers across different sectors, including meat, milk products, fruits, and vegetables. Thus, the motive of this review article is to offer a brief outline of (a) the source materials of marine biopolymers, which incorporates marine biopolymers derived from seaweed and marine animals, (b) a marine biopolymer classification, and (c) the various food applications in different food systems such as meat, milk products, fruits, and vegetables.

## 1. Introduction

The protection of food quality and the assurance of microbiological safety are the two most crucial aspects of food packaging. Beyond making it possible to handle, transport, and store a variety of food commodities, packaging also shields food from physical, chemical, and microbiological harm, improving quality preservation and shelf life. Long-chain hydrophilic polymers derived from polysaccharides and proteins are examples of biopolymers [1]. However, the preponderance of materials utilized in the packaging of food is polymeric polymers obtained from petroleum, also known as plastic [2]. The biodegradability, recycling, biocompatibility, and reusability of plastic packaging are hampered [3]. Polymeric materials separate the above from others for their advantages, such as flexibility and degradability, which allow the manufacturing of flexible pouches, bags, or stiff containers in a variety of shapes and sizes [4]. As a result, they are widely employed to hold and preserve fresh food commodities. This plastic packaging results in a mass of waste, which has a severe effect on the ecosystem by contaminating soil, polluting the ocean, polluting the air, and killing animals all over the world. The entire human habitat has now been poisoned by discarded plastics.

Nowadays, an upsurge is being noticed regarding the use of polymers made from renewable resources to create environmentally acceptable materials, using polysaccharides and proteins (such as starch, chitosan, and cellulose) which are directly obtained from biomass (including soy protein, caseinates, gluten, whey protein, and zein) [5]. Polyhydroxyalcanoates (PHAs) and bacterial cellulose are examples of microorganisms that can create polymers naturally or through genetic modification [6]. These biobased polymers are beneficial in three primary areas for use: as edible films for food, encapsulating/packaging of food, and as food-coating materials.

Marine sources are becoming more popular and desirable as cutting-edge resources for producing biopolymers such as proteins and polysaccharides [7]. These biopolymers have been promoted as being useful in the formation of packaging materials for food commodities due to their biocompatibility, biodegradability, and nontoxic properties. When marine resources such as mollusks, crabs, and fish are developed industrially to create commercial products, marine biopolymers undergo a significant development [8]. Muscle proteins, collagen, and gelatin are the three main categories of common marine proteins, whereas chitin, chitosan, alginate, agar, and carrageenan are examples of common marine polysaccharides. Then, using these proteins and polysaccharides, biodegradable films and coatings are created along with the necessary mechanical qualities to shield the food product inside from various spoiling factors. The ability of these packaging materials to decompose in the environment is their most significant quality and may also be the most anticipated.

Marine biopolymers are used in food to accomplish a variety of tasks, such as stabilizing foams, dispersions and emulsions thickening and gelling aqueous solutions, preventing the development of sugar and ice crystals, preventing degradation, and controlling the release of additives. The strong commercial position that marine biopolymers have recently attained has attracted numerous marine researchers and consumers [9]. However, before selecting a packaging application, numerous modifications are needed to make the coating material used as packaging material derived from marine biopolymers. For example, a material characterization of the packing material (such as its mechanical and barrier properties) is required to demonstrate its applicability for specific applications. In order to create sustainable and value-added goods, it may be worthwhile to value these abundant and accessible biowastes. Fish canning, lobster industry, and fisheries waste that has been landed are among the sources that are rich in biopolymers such as collagen, gelatin, chitin, and chitosan [10]. Shells from crabs and shrimp can be used to produce chitin in the case of products from mollusks and crustaceans. Chitin and collagen both have structural purposes. To expand its use, chitin is typically converted into chitosan, a water-soluble polymer [11].

So far, there have already been a few reports published on marine biopolymers. Uranga et al. (2021) studied the food-packaging applications of marine-originated biopolymers [12]. Polysaccharide-based marine biopolymers with multifunctional uses were reported by Ruocco et al. (2016) [13]. In another work, the food-packaging applications of marine biopolymers were studied by Mahmud et al. (2021) [14]. Although some reviews have been published on this topic, the application of marine biopolymers in various foods (milk, meat, fruits, and vegetables) has not been completely explored. Therefore, this review aimed to provide an overview of the sources of marine biopolymers and their classification. The application of marine biopolymers in various foods has also been discussed. The insight of this review is expected to provide a promising overview of marine biopolymers and their potential in various food applications.

The use of marine polysaccharides and proteins in the development of packaging materials, as well as their application in food-preservation methods, can provide valuable insights into sustainable solutions for the food-processing industry. Exploring the unique properties of marine biopolymers and their potential applications in different food systems, such as milk, meat, fruits, and vegetables, can shed light on their versatility and effectiveness.

This study highlights the classification of various sources of marine biopolymers. It is crucial for understanding the availability of marine biopolymers and their extraction methods. Different marine organisms, such as seaweed, algae, and fish, can serve as sources of these biopolymers, each with their own specific properties and characteristics. Highlighting the application of marine biopolymers in food-preservation methods, including films, coatings, and other techniques, can showcase their potential to enhance the shelf life and quality of food products. This can have significant implications for reducing food waste and improving sustainability in the food industry. The major challenges and proposed resolutions related to the use of marine biopolymers in food processing are also briefly provided in this review, which shows the practical insights for researchers and industry professionals. These challenges may include issues such as cost-effectiveness, scalability of production, and regulatory considerations. By addressing these challenges and proposing potential solutions, this study can contribute to the advancement and wider adoption of marine biopolymers in the food industry.

Lastly, exploring the future aspects and perspectives of marine-based biopolymers in food processing is crucial for understanding the potential growth and impact of this field. Identifying emerging trends, technological advancements, and potential areas of innovation can help researchers and industry stakeholders capitalize on the opportunities presented by marine resources for sustainable food-processing applications. Overall, this study has the potential to provide a comprehensive overview of the current state of marine biopolymers in the food-processing industry, as well as offer valuable insights into future directions and opportunities.

## 2. Sources of Marine Biopolymers

Seaweed and aquatic animals both contribute to the production of marine biopolymers, which are derived from two different sources [15]. Today, the diversity of species in the marine ecosystem plays a crucial role in many different industries [16].

### 2.1. Biopolymers Derived from Seaweed

**Figure 1:**
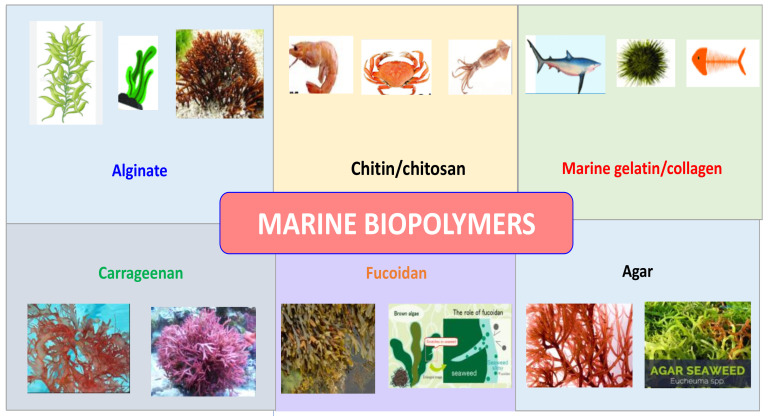
Biopolymers from marine sources.

Seaweed polysaccharides have gained increasing interest due to their abundant renewable sources and endearing qualities. There are around 8000 different varieties of seaweed in the ocean [17]. The marine macroalgae produce a variety of useful polysaccharides with prospective uses that serve as the cornerstone of the expanding blue economy. They can be used as functional ingredients for human health since they have potential uses in pharmaceuticals, nutraceuticals, and cosmetics [18]. Marine biopolymers derived from seaweed can be found in a variety of phyla, including phaeophytes or brown algae, rhodophytes or red algae, and chlorophytes or green algae (e.g., ulvan) [19]. More and more people are thinking about macroalgae as a possible source of bioactive substances as people become more aware of the functional components found in food [20]. Seaweed possesses significant potential as a renewable source of biopolymers which exhibit exceptional properties for a wide range of applications. These biopolymers have the ability to form films and demonstrate excellent mechanical characteristics. By employing various modification techniques such as reinforcement and blending, the desirable properties of these biopolymers can be further enhanced. The utilization of seaweed as a filler in polymer composites shows promising results in improving the thermal, physical, and mechanical properties of synthetic polymer matrices. In conclusion, seaweed represents a valuable renewable resource for the creation of eco-friendly materials that are biocompatible. Agar, a polysaccharide derived from seaweed, finds extensive use in microbiological media for its ability to provide a solid texture. It possesses specific qualities that make it suitable for coating meats [21]. Agar has the unique property of forming robust gels, which exhibit high melting points well above the initial temperature at which gelation occurs. Extensive research has been conducted on composites based on polysaccharides derived from seaweed, driven by their renewable and sustainable nature, particularly in industries such as food packaging and medical fields such as tissue engineering and drug delivery. Seaweed derivatives such as alginate, carrageenan, and agar are widely utilized in these applications due to their abundant availability, biocompatibility, and gelling capabilities. While seaweed exhibits notable film-forming characteristics, its mechanical strength and water-vapor-barrier properties are relatively limited. Therefore, modifications are essential to improve and enhance the properties of seaweed for these applications [22]. Brown algae, a diverse group of marine organisms, consist of over 250 genera and 1500–2000 species. These algae play a crucial role in coastal ecosystems as they are the primary producers of biomass in these regions. One of the notable characteristics of brown algae is the presence of fucose-containing sulfated polysaccharides in their cell walls. These sulfated polysaccharides, particularly a type of polysaccharide known as a fucoidan, are unique polymers that exhibit a wide range of properties and functions [23]. Algal-based polymers have found widespread application in various industries due to the high nutritional value of seaweed. These marine algae serve as the exclusive sources for important industrial phycocolloids, including agar, carrageenan, and alginate, which have multiple uses as stabilizers, viscosifiers, gelling agents, and emulsifying agents. Agar, carrageenan, and alginate are utilized as additives in a range of food products. They function as stabilizers, enhancing the texture, consistency, and shelf life of foods. These polymers contribute to the thickening, gelling, and emulsifying properties of various food items, such as sauces, dressings, desserts, ice creams, and dairy products [24]. The sources of various marine-originated biopolymers are schematically represented in Figure 1.

#### 2.1.1. Agar

Agar is constructed of two polysaccharides called agarose and agaropectin and is extracted from red seaweed [25]. Agar is composed of D-galactose and 3,6-anhydro-β-galactose with sulfated functional moieties. Agar helps create a thermo-reversible gel that becomes liquid at temperatures higher than 80 °C [26]. Agar contains a double-helix structure, which accumulates to form a 3D network which can hold water, giving agar its gelling capabilities. It has extensive use in the food industry, as well as in microbiology and the pharmaceutical industry [27].

The first phycocolloid to be utilized as a food additive in our civilization was agar–agar, generally known as just agar, and it extends back more than 300 years to the Far East. Phycocolloids are gelling substances obtained from seaweed used in several applications [28]. Agar first originated in Japan in 1658. It was first introduced in the Middle East and then expanded to the other nations that produce agarophyte seaweed. Its use was first observed in Europe in 1859, and bacterial-culture media began to contain it in 1882 [29].

Agar-gum-based continuous and translucent film is emerging as a famous and sustainable replacement for plastic-based food-packaging solutions [30]. Few researchers have attempted to investigate the usage of a composite film made of nanocellulose and seaweed extract (agarose) for packaging purposes [31]. The current work relates to the synthesis, processing, and characterization of agar-based biopolymer-film coatings on paper substrates in accordance with particular specifications established by the certifying bodies. It involves research on the mechanical characteristics of biopolymer films coated with agar, utilizing nanoindentation techniques, profilometry, and surface characteristics including the coating’s hydrophobicity and the film’s potential as a gas barrier [32]. The main ingredient of agar (seaweed extract), agarose, is a neutral polymer whose rheological qualities can be modified by adding glycerin or ethylene glycol. Additionally, it can be utilized as a biopolymer coating on a cellulose-extract paper substrate for applications in food packaging [33].

#### 2.1.2. Alginate

Alginate is a naturally occurring anionic polysaccharide that is mostly derived from brown seaweed. Because of its biocompatibility, nontoxicity, and mild gelation caused by the addition of many divalent cations such as Ca^2+^, alginate is most commonly used in the food and pharmaceutical industries [34]. Alginate is composed of two copolymers, guluronic acid and mannuronic acid. Alginate is a widely used polymer for encapsulation because it creates a flexible, nontoxic, and biocompatible gelled matrix capable of shielding the active ingredient from components such as heat and moisture, increasing stability [35]. Alginate has also drawn the attention of numerous researchers due to its biocompatibility, affordability, tolerable taste and smell, ease of handling, low toxicity, and moderate gelation qualities [36].

The primary commercial sources of alginate are considered to be brown seaweeds such as *Ascophyllum*, *Durvillaea*, *Ecklonia*, *Laminaria*, *Lessonia*, *Macrocystis*, and *Sargassum* [37,38]. This hydrocolloid, which may be produced at a minimal cost from coastal brown algae, has a potential application in the treatment of diabetes since it can lessen oxidative stress, improve insulin resistance, and reduce chronic inflammation, in addition to acting as an insulin-delivery system [39]. Supplements derived from an alginate source have been utilized as an adjuvant treatment to calorie restriction, to induce satiety, and to improve weight reduction in obese people; hence, it has also been acknowledged as a viable weight-loss treatment [40]. Alginate thus has promise as a useful substance utilized in both the food industry and in the treatment of metabolic illnesses including diabetes and obesity [41].

Sodium salt is the alginate with the greatest industrial significance. Along with alginic acid itself, uses are also identified for its potassium, ammonium, and calcium salts. Propylene glycol alginate is the only artificial alginic acid derivative that has gained widespread acceptance as a food additive. Stanford made the initial discovery of alginic acid [42,43]. Biologically superior and potentially therapeutic, alginate oligosaccharides (AOS) are produced from alginate [44]. Alginates made from the capsular polysaccharides of bacteria such as *Azotobacter vinelandii* and *Pseudomonas aeruginosa* are structurally and acetylated different from one another and therefore are not widely used in industrial settings [45]. For instance, alginate-based films, which are completely biodegradable and edible, have long been used to package water-soluble powder products including coffee, coffee-based treats, powdered milk, and instant teas [46]. Alginate is known as a food additive (E400) in the food sector.

#### 2.1.3. Carrageenan

Red algae cell walls contain a class of high-molecular-weight sulfated galactans known as carrageenan [47]. Carrageenan is made of alternate units of D-galactose and 3,6 anhydro-galactose joined by α-1,3 and β-1,4–glycosidic linkage. These polysaccharides’ structural variety results in a wide range of rheological qualities that are beneficial in both food and nonfood applications. In certain significant red algal families, carrageenan synthesis changes depending on the plant’s reproductive stage [40]. As a result, the different algal species exhibit varying carrageenan composition due to both genetic species specialization and other factors [48]. When the red algal species *Chondrus crispus* was utilized as a food-thickening agent and given the name “Irish moss”, it is believed that carrageenan from seaweed was first used several hundred years ago. The steps involved in producing carrageenan are extraction, purification, concentration, precipitation, and drying [49]. After gelatin and starch, carrageenan is the third most important hydrocolloid in the food industry [50]. Carrageenans are typically hydrophilic colloids that are soluble in water but insoluble in the majority of organic solvents. There are two types of carrageenans, semi-refined and refined [51].

In the manufacturing of semi-refined carrageenans, carrageenans are not taken out of the seaweed; instead, they are heated with an alkaline potassium hydroxide solution to about 75 °C [52]. Although potassium forms a bond to carrageenan and leads to the formation of a gel by limiting the dissolution of the hydrocolloid chains, to create κ- and ι- carrageenan, hydroxide combines with the sulphate esters, improving the gel strength of the finished product [53]. Refined carrageenan is created by heating seaweed fronds that have been alkali-treated (95–110 °C) to breakdown the gel matrix using the semi-refined carrageenan extraction method. Refined carrageenan extraction is substantially more expensive than semi-refined carrageenan processing [54]. Carrageenan is extracted using enzymes rather than chemicals because chemicals have a negative impact on the environment.

Carrageenan has been investigated as an active ingredient in the pharmaceutical industry for usage as a carrier/stabilizer in micro/nanoparticle systems, a novel extrusion aid for the production of pellets, and a polymer matrix in oral extended-release tablets, among other applications [55]. For many years, carrageenan has been utilized extensively as a food additive, and a substantial body of research supports its safety. Carrageenan is largely used in food processing to bind water and encourage the production of gels, thicken and solidify the structure of food items by tying up protein, and to enhance flavor [56]. A wide range of foods contain carrageenan, including dairy goods, water-dessert gels, meat and seafood, beverages, sauces, baby formulae, and pet food, among other things [57].

#### 2.1.4. Ulvan

Ulvans are sulfated heteropolysaccharides that are water-soluble and found in the cell walls of green marine macroalgae belonging to the genus Ulva. Ulva, commonly referred to as “sea lettuce”, is found all over the world and has been utilized in agriculture, medicine, and human sustenance [58]. One of the most prevalent genera of Chlorophyta, a class of seaweed grown in coastal locations, is Ulva. Ulva cell walls typically comprise two polysaccharide groups: the major group, which includes soluble ulvan and insoluble cellulose, and the minor group, which includes linear alkali-soluble xyloglucan and glucuronan [59]. Ulva has a high dietary-fiber content that supports digestive health and has been related to a decline in the prevalence of chronic disease [60]. Ulvan has a number of physiochemical and biological traits that could be useful in food, medicine, agriculture, and chemical applications [61]. Ulvan from green seaweed is highly sulfated because it contains rhamnose 3-sulfate, xylose, xylose 2-sulfate, glucuronic acid, and iduronic acid units [62]. Ulvan is a polysaccharide found in cell walls that makes about 9 to 36% of the dry weight of Ulva’s biomass. It is mostly made up of sulfated rhamnose, uronic acids (glucuronic acid and iduronic acid), and xylose [63]. These organic polymers are unique to green algae and are found in the genus Ulva [64]. With certain adjustments, ulvan polysaccharide can be extracted using the techniques of cold- or hot-water extraction and ethanol precipitation, and they can be utilized to create edible films (*Kappaphycus alvarezii* and *Ulva fasciata* [65]. The overall semi-refined extract yield of ulvan polysaccharide was 31.55% [66]

#### 2.1.5. Fucoidans

Fucoidan is a particular nomenclature term used by the International Union of Pure and Applied Chemistry (IUPAC) and is abundantly present in the cell-wall matrix of diverse types of brown algae. In both acidic and alkaline solutions, fucoidan is soluble. Sulfated polysaccharides, including fucoidan, have caught the interest of researchers among the various algal polysaccharides. They have a variety of biological effects, including antithrombotic, antiobesogenic, immunomodulatory, anticancer, and anticoagulant properties [67] The fucoidan polysaccharide is produced by brown macroalgae. Fucoidans can be found in large quantities in brown seaweed [68]. Fucose, a deoxyhexose sugar, is the primary subunit of fucoidan (C_6_H_12_O_5_) [69]. Fucoidan’s medicinal potential for cardiovascular disorders has received less research. The effects of fucoidan on the cardiovascular system have been shown in an increasing number of studies over the past few years [70]. Kylin originally isolated fucoidan from brown algae in 1913, it has been sold as a dietary supplement in the food business [71]. Fucoidan’s significant anticancer action has led to the most in-depth research in the field of cancer treatment [72]. Fucoidan has garnered interest due to its ability to offer a defense against liver and urinary system malfunctions [73]. Some Asian diets now include brown seaweeds that contain fucoidan, particularly in Japan, China, and South Korea. Fucoidan is nontoxic, rarely produces allergic reactions, and has a wide range of biological functions that are advantageous for therapeutic usage [74]. Pharmacologically, fucoidan influences numerous pathophysiological procedures, such as oxidative stress, inflammation, vascular physiology, and carcinogenesis [75]. According to reports, fucoidan affects various stages of inflammation, namely, (a) limiting lymphocyte adhesion and invasion, (b) inducing apoptosis, and (c) inhibiting a number of enzymes [76].

#### 2.1.6. Laminarins

Schmiedeberg was the first to isolate laminarin from the Laminariaceae, commonly known as laminaran or leucosin (1885) Brown algae contain laminarin, a low-molecular-weight polysaccharide and bioactive substance. It is found in the cell’s vacuoles. Fronds of *Laminaria* and *Saccharina* species contain laminarin [77]. The polysaccharide known as laminarin was created from the marine alga *Laminaria digitata* and sulfated with chlorosulfonic acid in pyridine and liquid sulfur dioxide at temperatures below 0° [78]. Additionally, laminarin possesses a few bioactive qualities that are of research interest and have potential commercial applications. Laminarin extraction from brown algae can be performed simultaneously with the extraction of the other polysaccharides found in the brown-algae cell wall, which helps to maximize the usage of the algal biomass. Laminarin is found in a variety of algae species, including *Ecklonia kurome*, *Laminaria japonica*, *Laminaria digitata*, and *Eisenia bicyclis.* Laminarin is a glucan that can be hydrolyzed into simple sugars such as glucose and other simple sugars, which microorganisms can then ferment to make ethanol as a byproduct [79]. Laminarin is primarily made up of neutral carbohydrates, with trace amounts of uronic acid [80] According to reports, it possesses pharmacological qualities such as being a neuroprotective potential antioxidant, anticancer, antitumor, immunomodulatory, anticoagulant, antiobesity, anti-inflammatory, antidiabetic, and wound healing. Due to its biodegradability, biocompatibility, and low toxic content, it has received extensive research as a useful material for biomedical applications [81].

### 2.2. Biopolymers Derived from Marine Animals

The enormous range of vertebrate and invertebrate species that live in settings ranging from the intertidal to deep water is how marine biodiversity is expressed. The amazing diversity of “forms and functions” displayed by marine species raises the possibility that they are a promising source of bioactive chemicals and can serve as an inspiration for various biomimetic techniques. Animals classified as aquatic refer to those that spend the majority of their lives in water bodies, such as lakes, rivers, ponds, seas, and so on, for example, jellyfish, sharks, fish, whales, sea otters, crabs, octopuses, mussels, dolphins, etc.

#### 2.2.1. Chitin and Chitosan

Chitin, a polysaccharide, is a part of the exoskeletons of crustaceans such as crabs and shrimp; in fact, crab shells are the main suppliers of chitin and chitosan. Chitin is one of the most prevalent marine biopolymers and has a number of uses in the biomedical and food industries [82]. The term “chitin” was reportedly first used by Bradconnot in 1811 and is derived from the Greek word “chiton”, which means a coat of mail [83]. Due to its biocompatibility, biodegradability, and bioabsorption, as well as its antibacterial and wound-healing properties and low immunogenicity, numerous papers on its biomedical applications have been published [84]. One of the most prevalent renewable biopolymers on earth, chitin can be obtained from marine sources for a reasonable price. Chitin is N-deacetylated to produce chitosan, and the amount of deacetylation varies depending on the kind of chitin [85]. As a result, a wide variety of applications have been documented in numerous industries including food technology, material science, microbiology, agriculture, wastewater treatment, drug-delivery systems, tissue engineering, and bionanotechnology. Numerous different organisms have been investigated for their chitin production. The chitin synthase (CS) enzyme is responsible for this production. CS I, CS II, and CS III are the three CSs of *Saccharomyces cerevisiae* [86]. Chitin is a polysaccharide, or more specifically, an amino glucopyranan, made up of glucosamine (GlcN) and N-acetylglucosamine (GlcNAc) units that are joined by covalent connections of (1, 4) [87]. Chitin can also be treated quickly to create nanofibers, gels, and membranes. Chitin and chitosan, which contain biological characteristics including anti-inflammatory, anticoagulant, antibacterial, and anticancer properties, have been extensively studied for the creation of desired biomaterials, particularly in the fields of tissue engineering, wound healing, and drug delivery [88]. Chitosan is used extensively to treat both acute and chronic wounds because of its ability to promote tissue regeneration and hemostasis [89]. Every year, more than 13,000,000 tons of crustaceans are harvested from marine environments worldwide, producing enormous volumes of food waste. The place of chitin’s origin affects the characteristics of chitosan. When removed, seaweed chitin is dense, solid, and insoluble [90]. Chitosan from marine-based sources is utilized in the food sector as a coating agent for the purpose of preservation, as well as an antioxidant and antibacterial agent [91]. Chitin and chitosan biodegradation are regulated by MW, pH, deacetylation level, and manufacturing technique. In reality, shellfish waste might provide more than 10,000 tons of chitin annually [92].

#### 2.2.2. Marine Collagen

A polypeptide called collagen is the most common protein in both animals and humans. In recent years, the interest in marine species as a safer, more flexible source of collagen has grown among researchers. The connective tissues of sea cucumbers, sea urchins, jellyfish, and starfish, among others, can also be used to separate marine collagen, which is often type I [93]. Significant interest has been shown in using fishing-industry waste as a sustainable and affordable source for collagen extraction. Due to its biocompatibility and good degradability, collagen can be used in a range of applications. Although marine collagens are both fibrillar and nonfibrillar, they have higher viscosities than corresponding bovine forms and lower gelling and melting temperatures than mammalian collagen. Compared to mammals, fish collagen has labile cross-connections that make it more susceptible to heat [94]. The majority of fish collagens have been discovered to include two chains, which are typically referred to as α-1 and -2 [95]. In the 1980s, electron microscopy was used for the first time to demonstrate the presence of collagen in both marine and freshwater sponges. In significant quantities, between 50 and 70%, waste materials such as skin, bones, fins, and scales are produced during the processing of fish. Due to their high collagen content, these waste products are becoming more popular as collagen sources [96]. The key component for obtaining collagen from underdeveloped fish resources may also be the waste from the processing of surimi. Several studies on the extraction of collagen from Pacific whiting surimi-processing byproducts, discarded fish skins, and fish waste materials have been published [97]. Collagens are used as food additives to verify that there are enough animal nutritional fibers present in sausages and frankfurters, as well as to improve the rheological properties of those foods [98]. Collagen has many applications in the food and beverage industries.

Collagen can be used as a collagen supplement, as a food additive, in films and coatings, in drinks, as carriers, etc. Collagen has raised its demand as a component for the development of nutritious foods. With aging and a poor diet, the body produces less collagen. The development of the collagen-supplement industry is a result of the association between collagen and health benefits. Collagen and its components have demonstrated a vital role as valuable nutritive fibers and protein sources in assembling human food because of its moisture-absorption properties. Collagen supplements are used to support the consumers’ body tissues, including their skin, hair, and nails [99]. Supplemental collagen can quicken lean muscle growth, shorten recovery times, repair damaged joint structures, and enhance cardiovascular performance [100]. Collagen films are primarily used as a barrier membrane to inhibit oxygen, moisture, and solute movement while also giving food goods structural integrity and vapor permeability. Additionally, edible films in food products have a great chance of extending a food’s shelf life.

#### 2.2.3. Marine Gelatin

Collagen is hydrolytically degraded and denaturized to produce gelatin. Gelatin consists of a large number of glycine, proline, and 4-hydroxy proline residues. In order to create environmentally friendly substitutes for synthetic ones, bioactive films made from marine gelatin are commonly used for food-packaging purposes. The demand for marine foods, particularly fish, has significantly expanded globally in recent years. The vast majority of collagen products on the market come from mammalian sources. Although fish-derived gelatin is a good substitute for mammalian-sourced gelatin, there are certain drawbacks to using it, including its smell, color, functional qualities, and uniformity in amino acid composition [101].

#### 2.2.4. Heparins and Heparan Sulfates

Heparins are polysaccharides found in the tissues of terrestrial and marine vertebrates and invertebrates. They are members of the glycosaminoglycan (GAG) family. The first anticoagulant to be used clinically was heparin. Ironically, McLean developed heparin in 1916 while trying to find a thromboplastic agent [102]. Heparan sulfates are produced by almost all cell types, whereas more sulfated heparins are mostly produced by mast cells [103]. To the best of our knowledge, there have not yet been any reports on heparins or heparan sulfates recovered from marine species as electrospun nanofibers.

## 3. Classification of Marine Biopolymers

**Figure 2:**
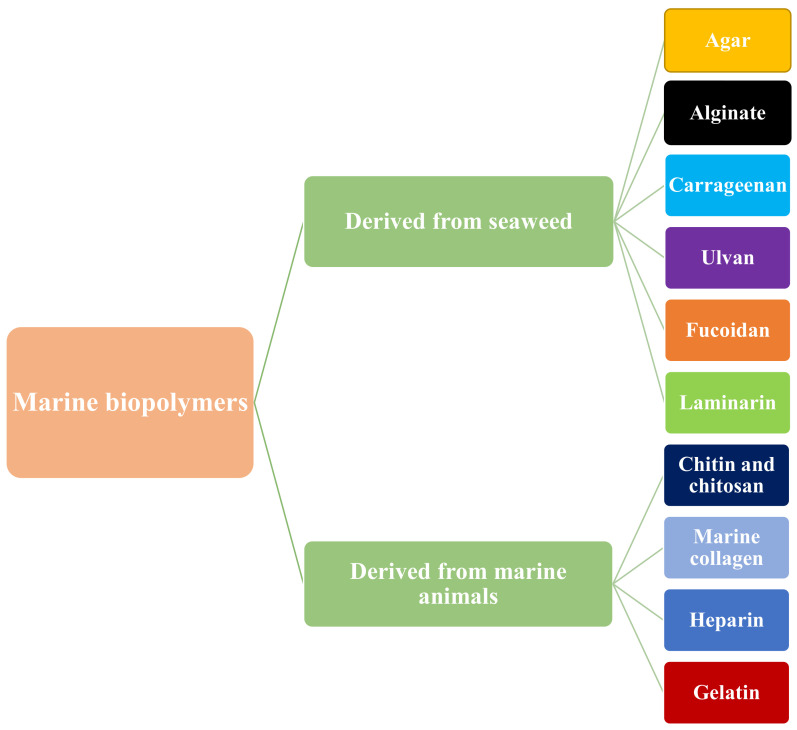
Classification of marine biopolymers.

Marine biopolymers are a diverse group of naturally occurring polymers that are found in marine organisms. They can be classified into three main categories: polysaccharides, proteins, and lipids. Polysaccharides are complex carbohydrates that are found in marine algae and seaweed. They are formed by repeating units of simple sugars and can be further classified into groups such as cellulose, alginate, and carrageenan. Proteins are large, complex molecules made up of amino acids. They are found in a wide variety of marine organisms, including fish and shellfish. Marine sources are becoming more well-known and in-demand as cutting-edge resources for producing biopolymers such as proteins and polysaccharides [104]. These biopolymers have been touted as useful in the creation of food-packaging materials because of their biocompatibility, biodegradability, and nontoxic properties [105]. The classification of marine-based biopolymers is given in Figure 2.

### 3.1. Polysaccharide-Based Marine Biopolymers

Marine biopolymers are a diverse group of naturally occurring polymers that are found in marine organisms. One of the most well-known and widely studied types of marine biopolymers are polysaccharides. Polysaccharides are complex carbohydrates that are composed of repeating units of simple sugars [106]. They are found in a wide variety of marine organisms, including algae and seaweed. One of the key characteristics of polysaccharides is their ability to form gels. This property is particularly useful in a number of industrial applications, such as food-thickening and gelling agents, and in the pharmaceutical industry for the formulation of drug-delivery systems. For example, alginate, a polysaccharide derived from brown seaweed, is widely used as a thickening and gelling agent in the food industry. It is also used in the biomedical field as a scaffold material for tissue engineering. Carrageenan, another polysaccharide derived from red seaweed, is widely used as a thickening agent in the food industry, as well as in the pharmaceutical industry for the formulation of oral and topical drugs. Another important application of marine polysaccharides is in the field of biotechnology [107]. Due to their structural diversity, polysaccharides are able to interact with a wide range of molecules and can be used as biosensors, bioactive agents, and in the production of biofuels. For example, cellulose, a polysaccharide found in marine algae, is a potential source of biofuel. Cellulose can be converted into glucose, which can then be fermented to produce ethanol.

Chitin is a biopolymer that is spontaneously created by a variety of different living things. Chitin can be found in many places, but shellfish waste, such as that from shrimp, crabs, and lobsters, is the most popular. A few types of fungi also include chitin [108]. The chitin’s place of origin affects the characteristics of chitosan. When removed, seaweed chitin is dense, solid, and insoluble. Chitosan created by deacetylating marine chitin is fantastic for packaging due to its high flexibility, reactivity, and water solubility [109]. All types of brown algae and some bacteria contain the marine biopolymer alginate, which gives their cell walls strength and flexibility [110,111,112] Red algae known as agarophytes are a subgroup of rhodophytes (red algae). In terms of agarophytes, the main commercial genera are *Gelidium*, *Pterocladiella*, *Gelidiella*, and *Gracilaria*. Agar is found in a variety of taxa, including *Ahnfeltia*, *Acanthopeltis*, *Campylaephora*, *Ceramium*, *Gracilariopsis*, and *Phyllophora* [113]. The conventional method for extracting agar includes the following steps: (1) soaking dry algae in boiling water to dissolve the agar gum from the cell wall; (2) screening the liquid extract; and (3) employing freezing and thawing operations to separate the gum from the water. Agar’s ability to gel could also be enhanced by using an alkali treatment, On the other hand, an alkali treatment might negatively impact the films [114,115,116]. Carrageenans are linear sulfated polysaccharide galactans that are isolated from species of red algae (Rhodophyta). After gelatin and starch, carrageenan is the third most important hydrocolloid in the food industry. The two most widely used marketable carrageenan are *Eucheuma denticulatum* and *Kappaphycus alvarezii*. Mostly, wild-harvested species of genera such as *Chondrus*, *Chondracanthus*, *Furcellaria*, *Gigartina*, *Iridaea*, *Mazzaella*, *Mastocarpus*, *Sarcothalia*, and *Tichocarpus* are used to make carrageenan raw materials. Carrageenan is manufactured in huge amounts worldwide, with the food industry accounting for 70% of its utilization [117,118]. The use of polysaccharide-based films and coatings in food packaging has significantly increased during the past few decades. In particular, marine polysaccharides with the capacity to shield food from contamination and deterioration, such as chitosan, alginate, agar, and carrageenan, have attracted a lot of attention in the creation of films and coatings in food packaging, boosting the shelf life and quality of foods [119].

### 3.2. Protein-Based Marine Biopolymers

Protein biomaterials have seen an increase in use recently in the food sector as a result of their superior gel-forming abilities, benign reactions, and biodegradable nature [120] Marine biopolymers are a diverse group of naturally occurring polymers that are found in marine organisms. One of the most important types of marine biopolymers are proteins. They are found in a wide variety of marine organisms, including fish, shellfish, and marine plants. Three crucial categories of muscle proteins derived from marine sources are myofibrillar, sarcoplasmic, and stromal proteins. About 65–75% of total muscle protein is made up of the contractile (myosin, actin) and regulatory (tropomyosin, troponin) myofibrillar proteins [121]. Muscle proteins, particularly those with high molecular weights such as myosin heavy chain, -actinin, have been reported to have been successfully extracted utilizing the nondenaturing method employing phosphate. In comparison to the denaturing process, the adoption of the nondenaturing extraction method lowers the release of hazardous and polluting substances while also saving time and labor. Because of their exceptional benefits, such as being UV-light barriers and good oxygen and carbon dioxide barriers, as well as their transparency, films and coatings made from muscle proteins have become a crucial component in the development of active packaging [122]. One of the most prevalent animal proteins, collagen can be found in all connective tissues, such as cartilage, tendons, ligaments, skin, and bones. Collagen has been shown to be abundant in marine sources, primarily fish byproducts, including sea urchins, starfish, and sea cucumbers [123]. Two essential processes are principally involved in the extraction of collagen: demineralization and raw-material pretreatment (alkaline and alcohol pretreatment to remove noncollagenous proteins and lipids, using ethylenediaminetetraacetic acid to remove calcium or other inorganic materials). Collagen can be extracted from marine sources using a variety of techniques, including those that use acid, salt, or enzymes to dissolve the collagen. Depending on elements such as fish age and type, the extraction procedure affects the ultimate yield and quality [124]. Collagen can be extracted and then subjected to hydrolysis to create gelatin. Collagen can be partially hydrolyzed and then heated to produce gelatin, a denatured protein [125]. Gelatin is an essential commercial biopolymer with numerous uses in the pharmaceutical and food-packaging industries. Certain chemical processes, such as deamination, esterification, acylation, interaction with acids and bases, and cross-linking, can aid to improve the rheological qualities of gelatin to improve its physical and chemical properties [126].

Another significant marine protein biopolymer, collagen, is mostly transformed into gelatin to create coatings and films appropriate for food-packaging applications. The most common application for marine collagen is in edible meat casings, where its ability to shrink and stretch allows it to simulate the expansion and contraction of meat batter during continuous processing [126]. Marine collagen is currently underutilized in food packaging, despite being used as a food ingredient to improve rheological properties. Collagen’s weak mechanical qualities and limited heat stability could be an issue. As a result, the majority of it is converted into gelatin, which is commonly used in the food industry.

Proteins play a vital role in the functioning of marine organisms, and they also have a wide range of industrial and biomedical applications. In the food industry, marine proteins are used as ingredients in a wide range of products, including fish and shellfish products, surimi, and protein concentrates. Marine proteins are also used as ingredients in animal feed and in the production of fertilizers and pesticides. In the biomedical industry, marine proteins have a wide range of applications [124]. For example, they are applied in the production of vaccines and diagnostic reagents, as well as in the formulation of new drugs. Marine proteins are also being researched for use in regenerative medicine and tissue engineering. For instance, collagen from fish skin and scales has been utilized in wound-healing applications. Another important application of marine proteins is in the field of biotechnology. Marine organisms, such as bacteria and algae, produce a wide range of enzymes that can be used in the production of biofuels, bioplastics, and other bioproducts. For example, enzymes from marine bacteria can be used to break down plant material, such as cellulose, for the production of biofuels. In conclusion, marine proteins are an important type of marine biopolymer that have a wide range of industrial and biomedical applications [125]. Their unique properties make them useful in the food, biomedical, and biotechnology industries. With ongoing research and development, the potential uses and applications of marine proteins are likely to continue to expand in the future.

One can infer, marine-based biopolymer sources are a natural factory of protein and carbohydrate biopolymers that can be used to create innovative food-packaging materials that can be groundbreaking in terms of bringing in money for the food industry, enhancing food safety, satisfying consumer demands, and lowering environmental issues imposed by many societies and institutions. With ongoing research and development, the potential uses and applications of marine polysaccharides are likely to continue to expand in the future.

## 4. Marine Biopolymers in Food Applications

Marine sources are becoming more well-known and sought-after as cutting-edge resources for the production of biopolymers such as polysaccharides and proteins. Because of their nontoxicity, biodegradability, and biocompatibility, these biopolymers have been hailed as valuable in the development of food-packaging materials. Several studies have thoroughly investigated the extraction, isolation, and covert use of marine biopolymers in the manufacture of environmentally acceptable packaging. Seaweed and marine creatures are abundant sources of marine biopolymers, which have a wide range of structural identities and functions as well as a wide range of beneficial biological activities [127]. The different types of marine biopolymers and their application in different varieties of food commodities is mentioned below in Table 1.

This review investigates the usage of biopolymers for innovative applications in a variety of critical sectors. Biobased polymers are becoming increasingly practical in terms of processing and cost for a wide range of unique industrial applications. They provide fresh potential for the development of biobased materials as raw materials, in response to recent demands for biodegradable plastic materials to reduce plastic pollution in marine ecosystems. Marine biopolymers have been addressed to have a diverse range of bioactivity. Chitosan and gelatin derived from marine biopolymer sources are useful both separately and in combination for preservation. To create active packaging with better quality, various elements (antimicrobial and antioxidant) are incorporated into films made of chitosan and gelatin [143]. These marine biopolymers are particularly appealing to the scientific community and a number of industrial sectors due to their enormous variety, renewable nature, tunable physicochemical and structural behavior, and appealing biological features. Biopolymers are a varied and adaptable family of materials with potential use in almost every sector of the economy. Gelatin and chitosan are commercially accessible marine biopolymers that are nontoxic, unscented, edible, biodegradable, and biocompatible. This makes them a suitable raw material in food-packaging applications. For example, an active film from chitosan and gelatin showed good physical properties such as mechanical properties, water vapor permeability, flexibility and oxygen permeability. Chitosan-based active films also exhibit microbial inactivation and oxidative-activity inhibition [144].

**Figure 3:**
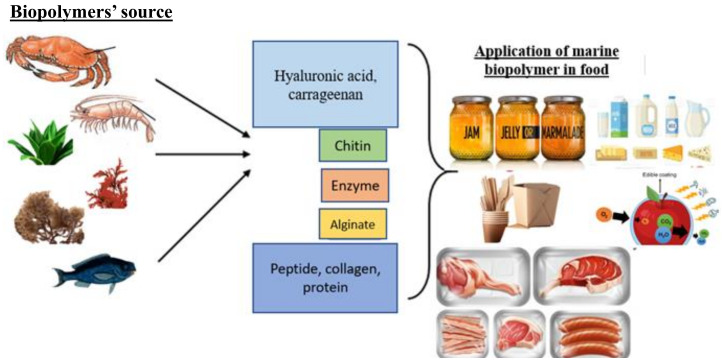
Application of marine biopolymers in different food systems.

Biopolymer applications are prevalent in many different fields where sustainable and biodegradable solutions are required, and biopolymers have piqued the interest of many people. Biopolymers have good potential in food applications. Marine biopolymers, because of their unique properties and ability to interact with other food components, have traditionally played a significant role in food processing. Chitosan and gelatin of 10% and 1%, respectively, were applied as spray coatings on vacuum-packaged beef to see how well they affected the shelf life of the product for up to 21 days, and the results showed that the coatings significantly reduced the rate of lipid oxidation and inhibited the growth of lactic-acid bacteria [145]. Natural pigments derived from marine biopolymer sources have caught the interest of the food and beverage industries due to their benefits and biological activities, such as possessing antibacterial, antioxidant, and sensory properties. They are commonly employed as a functional bioactive ingredient in food processing and packaging application [146]. Biopolymers are natural macromolecules that can be used to create environmentally friendly products with the purpose of long-term development. They are highly biocompatible, ecologically friendly, and can be used as starting reagents/modifiers in a variety of processes. Biomacromolecules/polymers’ physical and mechanical qualities are inadequate for direct industrial uses, particularly in packaging and product compositions. As a result, they are expected to undergo certain adjustments or to develop new inventive approaches to produce value-added industrial-grade biopolymers [147]. The application of marine biopolymers in various food applications is schematically shown in Figure 3.

### 4.1. Fish and Meat Products

Consumers’ desire for healthier eating has led to the development of naturally preserved meat products by meat scientists. Marine biopolymers with natural, antibacterial, and antioxidant properties have grown in popularity in the field of meat technology due to their long-term effectiveness on the product’s surface, resulting in the delay or suppression of chemical and microbiological deterioration [148]. Chitosan coating has been used successfully to reduce lipid oxidation (traditional dry-fermented sausage), lengthen the shelf life of fresh shrimp and ready-to-cook meat products (chicken balls, chicken seekh kababs, and mutton seekh kababs), and control *Salmonella* on fresh chicken breasts. Aroma scavengers/absorbers are designed to remove unwanted flavors, smells, and odors from the packaging headspace. These off-flavors and off-odors in food are primarily caused by the oxidation of lipids and oils, which results in the creation of aldehydes and ketones, or the breakdown of proteins from fish muscles to amines. It has been reported that chitosan films reduced both primary and secondary lipid oxidation [149]. Muscle protein biopolymers have a significant potential for food packaging [150]. The biodegradable films formed using fish myofibrillar and Whitemouth croaker residual protein isolates attained low water-vapor permeability and tensile strength [151].

Polysaccharides from *Spirulina platensis* are commonly used as a food ingredient or coloring agent in a variety of foods, including ice cream, chewing gum, candy, popsicles, dairy products, soft beverages, and jellies [152]. In food, these biopolymers’ gel works in thickening aqueous solutions, as well as stabilizing foams, emulsions, and dispersions, limiting ice and sugar crystal formation, preventing spoiling, and controlling the release of additional components. These food biopolymers have a significant impact on food structure, functional characteristics, processing, and shelf life. Because of the huge number of hydroxyl groups, they are generally hydrophilic in nature and have a high affinity for attaching to water molecules. Allowing them to be distributed in water in the colloidal state [153]. To optimize the process design, a response-surface methodology was used, yielding a bioplastic which is suitable for food packaging due to its flexibility, low solubility, mechanical strength, and water vapor permeability. It contains 0.79% protein (m/v) and 40% plasticizer (m/m). The presence of sulfhydryl groups on the surface of the myofibrillar protein, which permitted the production of covalent S-S in the biofilm framework, was credited with the high tensile strength (4.91 MPa). Yet the polarity of fish muscle proteins is what causes them to be hydrophilic. The bioplastic’s poor moisture barrier was due to amino acids and hydroxyl (OH) groups. In various food situations, gelatin-based coatings and films have been used to enhance the quality grade and durability of packed goods. For example, gelatin (0–6%) was combined with chitosan (0.5–1.5%) to create a film to preserve beef steak throughout five days of retail display. The mixed film was found to be beneficial at reducing weight loss, preventing lipid oxidation, and improving the color of the beef steak [154]. A team of researchers revealed that a film developed using chitosan and apricot-kernel essential oil greatly reduced fungal growth on packed bread slices. Furthermore, the modified films demonstrated an improved water resistance and water-vapor property. According to the findings, the created film contains antibacterial and antioxidant qualities and could be a good method for extending the shelf life of bread for better preservation [155].

### 4.2. Fruits and Vegetables

In recent years, a great variety of marine-derived biopolymers have been employed in conjunction with byproduct fractions to generate enhanced food-processing applications. Fruits and vegetables are crucial in the human diet because of the nutritional and health benefits they provide. However, they have a relatively short postharvest life since they continue as living tissues until they are consumed and are susceptible to physiological and biochemical changes, which can also have physical or pathological origins, resulting in significant economic losses [156].

Among the treatments, the fruit combination of heat treatment and chitosan coating showed the least amount of membrane permeability, ethylene generation, respiration rate, and malondialdehyde, as well as the maximum strength and consumer appeal [157]. Edible coatings are a conservation strategy that strives to meet current market demands while also being a straightforward process to implement when compared to other food-preservation methods. The food industry is continually striving to meet the requirements of consumers since changes in lifestyle necessitate changes in the accessibility of various goods, such as minimally processed vegetables and fruits. Much research has been conducted to support the possible significance of chitosan coating in extending the storage life and controlling the deterioration of fruits and vegetables. Chitosan is recognized to have plant-immunity-eliciting characteristics against several pathogens, and elicitation defenses account for 30 to 40% of total activity, which is significant in minimizing postharvest deterioration of fresh produce [158]. The use of edible coatings has been extensively researched in the past, but researchers’ interest has expanded with the rising demand for fresh-cut fruits and vegetables. Biopolymer-based edible coatings are a new packaging advancement for extending the shelf life of fresh-cut fruits and vegetables [159]. The films and coatings form a barrier environment that prevents the transmission of gases, oxygen, vapors, and other contaminants, consequently increasing the shelf life and quality features of packed fruits and vegetables. However, before being used in a packaging application, the functionality of the films and coatings generated from marine biopolymers requires certain changes [160]. The thermal stability and film thickness of green grapes incorporated with an agar–zinc-oxide-nanoparticle film are excellent. The packaged green grapes kept their original fresh appearance up to two weeks in ambient settings. The mechanical properties, thermal stability, and water vapor barrier properties of the nanocomposite films were all enhanced. Green grapes coated in prepared films retained their appearance after long durations of storage at room temperature. As a result, the newly developed nanocomposite film could be used as an excellent product-packaging material for prolonging the shelf life of green grapes [161]. Finally, when employed in conjunction with the concepts of active, intelligent, and green product technologies, marine biopolymers can produce a multifunctional food-processing system for fruits and vegetables, which is the ultimate goal of current food-processing technology.

### 4.3. Milk and Milk Products

Marine biopolymers have been incorporated to improve the texture, rheology, physicochemical qualities, and sensory attributes of dairy formulations in order to fulfil customer needs. Marine biopolymers may also increase the storage life of dairy-based products by minimizing wheying off, suspending dispersed particles, or suppressing protein micelle flocculation [162]. “Ayran”, a Turkish yoghurt beverage, generally consists of an increased rate of the flocculation of casein micelles, due to which the viscosity of the drink is decreased. Guar gum and high-methoxyl pectin have been incorporated to lessen the flocculation, thus making the drink more viscous [163]. Another study found that gum tragacanthin adhered to the casein particles in an Iranian yoghurt beverage called “Doogh” and inhibited the casein particles from aggregating by steric stabilization [164]. The most common marine hydrocolloid gelling agents include alginate, carrageenan, pectin, gellan, agar, and gelatin. Before creating gels, hydrocolloids are often used as thickening, stabilizing, or emulsifying agents in dairy products [153].

Several studies have shown that *Pseudomonas* spp. growing on the cheese’s exterior, primarily from water used during manufacturing, spoils mozzarella cheese quite frequently. Another factor reducing the perishable life span of mozzarella cheese is the growth of coliforms. At the moment, the shelf life of mozzarella cheese is roughly 5 to 7 days and attempts are being made to extend this shelf life through innovative processes and raw-material quality enhancement. Because it is ecologically friendly and very inexpensive, the application of chitosan as an antibacterial agent to extend the shelf life of packaged mozzarella cheese could be feasible [165]. This is due to the fact that chitosan is a deacetylated version of chitin, the second most prevalent biopolymer on the planet after cellulose [166]. Chitosan, sodium alginate, and a sweetener (fructose) were used in the preparation of dairy desserts. It was discovered that the produced milk desserts had a preventive role against erosive and ulcerative lesions generated by an aspirin-stomach-injury model, which allows us to classify a product to a functional food group [167]. Yogurt is an extremely popular dairy byproduct among consumers, as well as one of the most significant sources of good lactic-acid bacteria. Alginate can be used as a stabilizer in yoghurt products with a pH range of 3.9–4.9. For these items, the optimum stabilizer is propylene glycol alginate. When mixed, frozen buttermilk stabilized with alginate has a decent consistency and no stickiness or stiffness. Alginate can also help to keep yoghurt viscosity stable after sterilization [168].

Gelatin is a biopolymer generated by partial collagen hydrolysis or heat denaturation. Gelatin is mostly made from byproducts of land-animal and fish processing, such as skin, bones, tendons, scales, and so forth. It frequently appears in both food products to improve stability, flexibility, and consistency. Gelatin raises the viscosity of cream and reduces the time required to whip it. It also improves foam stability, resulting in excellent over-run properties and a very light silky texture [169]. Because of the high temperatures used during pasteurization, which is used in these blended cheese products, most of the milk protein results in denaturation that leads to the loss of the natural water-binding property that must be restored. The inclusion of 0.5 to 0.8% gelatin reduces syneresis, and the resulting gel improves spreading ability while giving a smooth body. Gelatin, on the other hand, elevates the viscosity of cottage cheese dressing, resulting in a creamed curd with no loose cream or whey, as well as a uniform dispersion of the dressing throughout the product. In fact, the maker can minimize the fat percentage while still producing a delicious, creamed cottage cheese [170]. As a result, the application of marine based biopolymers in the production of dairy products is currently very promising because it enables the creation of innovative functional goods with therapeutic properties.

## 5. Conclusions and Future Perspective

A viable substitute for synthetic polymers made from petroleum is the wide variety of biodegradable and sustainable biopolymers. One of the key origins of biopolymers is marine resources. Agar, alginate, carrageenan, chitosan, and gelatin are the most used and relevant marine biopolymers in food applications. Through engineering improvements and research, sustainable alternatives to synthetic polymers have evolved, providing a workable solution to current environmental, social, and economic concerns. These biobased substitutes provide a more practical and sustainable solution than synthetic polymers made from petrochemicals. Marine-derived biopolymers are generally employed in a different industry, but the food industry has shown a particular interest. Due to their beneficial qualities as emulsifiers, gelling agents, thickening agents, and stabilizers, marine biopolymers are well used in the food-processing sector. Marine biopolymers with the necessary gas-barrier and heat-sealing qualities can produce excellent biocompatible films, which is advantageous for active and intelligent food-packaging applications. By incorporating appropriate nanofillers or bioactive molecules into the matrix of a marine biopolymer, the physical and functional characteristics of the film can be improved.

This review article explores the multifaceted role of the oceans as a source of inspiration and raw materials for the development of innovative materials. It highlights two main aspects: the utilization of biomolecules derived from aquatic organisms as biotechnological tools and the creation of materials that draw inspiration from marine principles, aiming to address pressing societal and environmental challenges such as pollution and the disposal of hazardous materials. The current review showcases the opportunities to develop novel materials that can contribute to solving critical global issues. It underscores the importance of harnessing the potential of the oceans for sustainable and innovative solutions in multiple industries.

Further studies are required to more fully explore the valorization aspects, mode of action of active ingredients, innovative means of enhancing technological tools, and cost competitiveness in order to establish a sustainable food-processing system using marine biopolymers. On the other hand, even if the use of marine biopolymers in the food industry appears safe and promising, there is still a need for further study to be conducted in order to make it available for products with additional value on the market. In addition, there is a concern linked with the toxicity of employing marine biopolymers because of the major threat of heavy-metal, chemical, and plastic contamination in the marine environment. While extracting marine biopolymers, a thorough purification procedure must be followed. The potential for using marine-derived materials as prospective biopolymers is still vast and expanding.

## Figures and Tables

**Table 1:** Application of various types of marine biopolymers in different food systems.

Biopolymer-Based Film/Coating	Food System	Key Outcome	References
Gelatin–chitosan film	Rainbow trout	The shelf life of rainbow trout fillets was extended by the application of a chitosan–gelatin composite film to over 16 days in refrigerated storage conditions, which reduced bacterial contamination.	[128]
Chitosan	Chicken	Peanut-skin-extract-added chitosan-based film decreased psychrotrophic microbial growth and improved the oxidative stability of the chicken product.	[129]
Alginate	Shiitake mushroom	Inhibition of the growth of mesophilic, psychrophilic pseudomonas and yeasts and molds.	[130]
Agar	Hake fillet	A green tea probiotic strain was added to the agar-based biopolymer film, which eventually delayed the growth of microbes and decreased the spoilage indexes.	[131]
Alginate	Strawberry	Strawberry coatings were an effective way to sustain water loss while significantly lowering solid gain under the studied osmotic dehydration conditions.	[132]
Chitosan and oxidized starch	Papaya	The shelf life of the coated fruit was extended. At room temperature, untreated papayas reached the point of ripening after 5 days, whereas coated papayas reached this point after 15 days, indicating that the coating helped to increase papaya pulp hardness.	[133]
Chitosan/lactic acid solution	Cheese	A chitosan/lactic acid solution was added in the starter culture; during refrigeration, it prevented the growth of rotting microorganisms for up to 10 days.	[134]
Chitosan modified by antimicrobial monomethyl fumaric acid (MFA)	Beef	Chitosan derivatives reduced the total viable count of lactic-acid bacteria yeast–mold, and psychrotrophic bacteria. Also, application of this derivative increased the shelf life by 8 days.	[135]
Chitosan	Fish oil	Free-radical-scavenging activity was increased as compared to the control group, resulting in increased storage life.	[136]
Gelatin	Cold-smoked sardines	Gelatin film enriched with oregano and rosemary helped lower the oxidation rate and increased the days of storage.	[137]
Chitosan and gelatin	Black grapes	The developed film extended the shelf life of the black grapes to up to 14 days during storage at 37 °C.	[138]
Alginate	Fresh-cut apples	The coating conferred increased shelf life by giving apples a good appearance and firmness, inhibiting enzymatic actions of browning, and reducing weight loss.	[139]
Agar	Green grapes	The zinc-oxide-added agar-based functional packaging film can be a promising active packaging material. The functional film could significantly improve the shelf life of green grapes.	[140]
Gelatin/agar	Pork	The clove essential oils and copper-doped zinc-oxide-included film were effective in reducing the lipid peroxidation and total microbial count in functional-film-wrapped pork. The shelf life of the meat was extended after the application of the packaging.	[141]
Gelatin/chitosan	Beef	The application of a gelatin and chitosan spray coating on the vacuum-packed beef enhanced the life span up to three weeks compared to the uncoated counterparts.	[142]

## Data Availability

Not applicable.

## References

[1] Tibolla H., Pelissari F.M., Martins J.T., Lanzoni E.M., Vicente A.A., Menegalli F.C., Cunha R.L. (2019). Banana Starch Nanocomposite with Cellulose Nanofibers Isolated from Banana Peel by Enzymatic Treatment: In Vitro Cytotoxicity Assessment. Carbohydr. Polym..

[2] Rai P.K., Choure K. (2023). Agriculture waste to bioplastics: A perfect substitution of plastics. Value-Addition in Agri-Food Industry Waste Through Enzyme Technology.

[3] Juikar S.K., Warkar S.G. (2023). Biopolymers for Packaging Applications: An Overview. Packag. Technol. Sci..

[4] Oyervides-Muñoz E., Oyervides-Muñoz M.A., Garcia-Lobato M.A. (2023). Chitin and chitosan nanocomposites: From the synthesis to the application. Green-Based Nanocomposite Materials and Applications.

[5] Grzebieniarz W., Biswas D., Roy S., Jamróz E. (2023). Advances in Biopolymer-based Multi-layer Film Preparations and Food Packaging Applications. Food Packag. Shelf Life.

[6] Moradali M.F., Rehm B.H.A. (2020). Bacterial Biopolymers: From Pathogenesis to Advanced Materials. Nat. Rev. Microbiol..

[7] Mahmud N., Islam J., Tahergorabi R. (2021). Marine Biopolymers: Applications in Food Packaging. Processes.

[8] Vera M., Mella C., García Y., Jiménez V.A., Urbano B.F. (2023). Recent Advances in Tannin-Containing Food Biopackaging. Trends Food Sci. Technol..

[9] Ruocco N., Costantini S., Guariniello S., Costantini M. (2016). Polysaccharides from the Marine Environment with Pharmacological, Cosmeceutical and Nutraceutical Potential. Molecules.

[10] Uranga J., Zarandona I., Andonegi M., Guerrero P., de la Caba K. (2021). Biopolymers derived from marine sources for food packaging applications. Sustainable Food Packaging Technology.

[11] Chakraborty K. (2023). Recent Advances in Marine Biotechnology. Frontiers in Aquaculture Biotechnology.

[12] Roy S., Zhang W., Biswas D., Ramakrishnan R., Rhim J.-W. (2023). Grapefruit Seed Extract-added Functional Films and Coating for Active Packaging Applications: A Review. Molecules.

[13] Nehra A., Biswas D., Siracusa V., Roy S. (2022). Natural Gum-based Functional Bioactive Films and Coatings: A Review. Int. J. Mol. Sci..

[14] Roy S., Siracusa V. (2023). Multifunctional Application of Biopolymers and Biomaterials. Int. J. Mol. Sci..

[15] Duarte C.M., Bruhn A., Krause-Jensen D. (2022). A Seaweed Aquaculture Imperative to Meet Global Sustainability Targets. Nat. Sustain..

[16] Westlake J.R., Tran M.W., Jiang Y., Zhang X., Burrows A.D., Xie M. (2023). Biodegradable Biopolymers for Active Packaging: Demand, Development and Directions. Sustain. Food Technol..

[17] Benjamin T.-A., Ahmad I., Sadiq M.B. (2023). Applications of marine biochemical pathways to develop bioactive and functional products. Marine Biochemistry.

[18] Mistry P.A., Konar M.N., Latha S., Chadha U., Bhardwaj P., Eticha T.K. (2023). Chitosan Superabsorbent Biopolymers in Sanitary and Hygiene Applications. Int. J. Polym. Sci..

[19] Yang Y., Zhang M., Alalawy A.I., Almutairi F.M., Al-Duais M.A., Wang J., Salama E.-S. (2021). Identification and Characterization of Marine Seaweeds for Biocompounds Production. Environ. Technol. Innov..

[20] Sudhakar M.P., Kumar B.R., Mathimani T., Arunkumar K. (2019). A Review on Bioenergy and Bioactive Compounds from Microalgae and Macroalgae-Sustainable Energy Perspective. J. Clean. Prod..

[21] Xue W., Zhu J., Sun P., Yang F., Wu H., Li W. (2023). Permeability of Biodegradable Film Comprising Biopolymers Derived from Marine Origin for Food Packaging Application: A Review. Trends Food Sci. Technol..

[22] Bukhari N.T.M., Rawi N.F.M., Hassan N.A.A., Saharudin N.I., Kassim M.H.M. (2023). Seaweed Polysaccharide Nanocomposite Films: A Review. Int. J. Biol. Macromol..

[23] Deniaud-Bouët E., Hardouin K., Potin P., Kloareg B., Hervé C. (2017). A Review about Brown Algal Cell Walls and Fucose-Containing Sulfated Polysaccharides: Cell Wall Context, Biomedical Properties and Key Research Challenges. Carbohydr. Polym..

[24] Azeem M., Batool F., Iqbal N., Zia K.M., Zuber M., Ali M. (2017). Chapter 1—Algal-based biopolymers. Algae Based Polymers, Blends, and Composites.

[25] Pandya Y.H., Bakshi M., Sharma A., Pandya H. (2022). Agar-Agar Extraction, Structural Properties and Applications: A Review. Pharma Innov. J..

[26] Glicksman M. (2019). Red Seaweed Extracts (Agar, Carrageenans, Furcellaran). Food Hydrocolloids.

[27] Zhang Y., Fu X., Duan D., Xu J., Gao X. (2019). Preparation and Characterization of Agar, Agarose, and Agaropectin from the Red Alga Ahnfeltia Plicata. J. Oceanol. Limnol..

[28] Pereira L. (2016). Edible Seaweeds of the World.

[29] Stefanowska K., Woźniak M., Dobrucka R., Ratajczak I. (2023). Chitosan with Natural Additives as a Potential Food Packaging. Materials.

[30] Mostafavi F.S., Zaeim D. (2020). Agar-Based Edible Films for Food Packaging Applications—A Review. Int. J. Biol. Macromol..

[31] Jumaidin R., Sapuan S.M., Jawaid M., Ishak M.R., Sahari J. (2018). Seaweeds as Renewable Sources for Biopolymers and Its Composites: A Review. Curr. Anal. Chem..

[32] Matheus J.R.V., Dalsasso R.R., Rebelatto E.A., Andrade K.S., de Andrade L.M., de Andrade C.J., Monteiro A.R., Fai A.E.C. (2023). Biopolymers as Green-based Food Packaging Materials: A Focus on Modified and Unmodified Starch-based Films. Compr. Rev. Food Sci. Food Saf..

[33] Roy S., Rhim J.-W. (2019). Agar-based Antioxidant Composite Films Incorporated with Melanin Nanoparticles. Food Hydrocoll..

[34] Roy S., Rhim J.-W. (2019). Melanin-mediated Synthesis of Copper Oxide Nanoparticles and Preparation of Functional Agar/CuO NP Nanocomposite Films. J. Nanomater..

[35] Venugopal V., Sasidharan A. (2021). Seafood Industry Effluents: Environmental Hazards, Treatment and Resource Recovery. J. Environ. Chem. Eng..

[36] Gupta V., Biswas D., Roy S. (2022). A Comprehensive Review of Biodegradable Polymer-Based Films and Coatings and Their Food Packaging Applications. Materials.

[37] Roy S., Rhim J.-W. (2020). Effect of CuS Reinforcement on the Mechanical, Water vapor barrier, UV-light barrier, and Antibacterial Properties of Alginate-based Composite Films. Int. J. Biol. Macromol..

[38] Rosadiani D.W., Purwanti T., Purwanto D.A. (2018). Effect of Natrium Alginate Concentration on Physical Characteristics, Viability and Anticancer Activity of Microparticles from a Combination of Probiotics and Tomato Pasta. Res. J. Pharm. Technol..

[39] Kanokpanont S., Yamdech R., Aramwit P. (2018). Stability Enhancement of Mulberry-Extracted Anthocyanin Using Alginate/Chitosan Microencapsulation for Food Supplement Application. Artif. Cells Nanomed. Biotechnol..

[40] Dufrane D., Goebbels R.-M., Gianello P. (2010). Alginate Macroencapsulation of Pig Islets Allows Correction of Streptozotocin-Induced Diabetes in Primates up to 6 Months without Immunosuppression. Transplantation.

[41] Chee S.-Y., Wong P.-K., Wong C.-L. (2011). Extraction and Characterisation of Alginate from Brown Seaweeds (Fucales, Phaeophyceae) Collected from Port Dickson, Peninsular Malaysia. J. Appl. Phycol..

[42] Ali S.W., Bairagi S., Banerjee S., Banerjee S. (2022). Plant and marine-based biopolymers for efficient nutrient delivery. Biopolymers in Nutraceuticals and Functional Foods.

[43] Vasudevan U.M., Lee O.K., Lee E.Y. (2021). Alginate Derived Functional Oligosaccharides: Recent Developments, Barriers, and Future Outlooks. Carbohydr. Polym..

[44] Nguyen B.V.G., Nagakubo T., Toyofuku M., Nomura N., Utada A.S. (2020). Synergy between Sophorolipid Biosurfactant and SDS Increases the Efficiency of P. Aeruginosa Biofilm Disruption. Langmuir.

[45] Gheorghita Puscaselu R., Lobiuc A., Dimian M., Covasa M. (2020). Alginate: From Food Industry to Biomedical Applications and Management of Metabolic Disorders. Polymers.

[46] Robal M., Brenner T., Matsukawa S., Ogawa H., Truus K., Rudolph B., Tuvikene R. (2017). Monocationic Salts of Carrageenans: Preparation and Physico-Chemical Properties. Food Hydrocoll..

[47] Roy S., Rhim J.-W. (2019). Preparation of Carrageenan-based Functional Nanocomposite Films Incorporated with Melanin Nanoparticles. Colloids Surf. B Biointerfaces.

[48] Dong M., Xue Z., Liu J., Yan M., Xia Y., Wang B. (2018). Preparation of Carrageenan Fibers with Extraction of Chondrus via Wet Spinning Process. Carbohydr. Polym..

[49] Nguyen P.T.M., Kravchuk O., Bhandari B., Prakash S. (2017). Effect of Different Hydrocolloids on Texture, Rheology, Tribology and Sensory Perception of Texture and Mouthfeel of Low-Fat Pot-Set Yoghurt. Food Hydrocoll..

[50] Qiu S.-M., Aweya J.J., Liu X., Liu Y., Tang S., Zhang W., Cheong K.-L. (2022). Bioactive Polysaccharides from Red Seaweed as Potent Food Supplements: A Systematic Review of Their Extraction, Purification, and Biological Activities. Carbohydr. Polym..

[51] Gupta I., Cherwoo L., Bhatia R., Setia H. (2022). Biopolymers: Implications and Application in the Food Industry. Biocatal. Agric. Biotechnol..

[52] Skryplonek K., Henriques M., Gomes D., Viegas J., Fonseca C., Pereira C., Dmytrów I., Mituniewicz-Małek A. (2019). Characteristics of Lactose-Free Frozen Yogurt with κ-Carrageenan and Corn Starch as Stabilizers. J. Dairy Sci..

[53] Martiny T.R., Pacheco B.S., Pereira C.M.P., Mansilla A., Astorga–España M.S., Dotto G.L., Moraes C.C., Rosa G.S. (2020). A Novel Biodegradable Film Based on Κ-carrageenan Activated with Olive Leaves Extract. Food Sci. Nutr..

[54] Zia K.M., Tabasum S., Nasif M., Sultan N., Aslam N., Noreen A., Zuber M. (2017). A Review on Synthesis, Properties and Applications of Natural Polymer Based Carrageenan Blends and Composites. Int. J. Biol. Macromol..

[55] Necas J., Bartosikova L. (2013). Carrageenan: A Review. Vet. Med..

[56] Gopi S., Balakrishnan P., Brai M. (2022). Biopolymers in Nutraceuticals and Functional Foods.

[57] Yu-Qing T., Mahmood K., Shehzadi R., Ashraf M.F. (2016). Ulva Lactuca and Its Polysaccharides: Food and Biomedical Aspects. J. Biol. Agric. Healthc..

[58] Madany M.A., Abdel-Kareem M.S., Al-Oufy A.K., Haroun M., Sheweita S.A. (2021). The Biopolymer Ulvan from Ulva Fasciata: Extraction towards Nanofibers Fabrication. Int. J. Biol. Macromol..

[59] Kidgell J.T., Magnusson M., de Nys R., Glasson C.R.K. (2019). Ulvan: A Systematic Review of Extraction, Composition and Function. Algal Res..

[60] Lakshmi D.S., Sankaranarayanan S., Gajaria T.K., Li G., Kujawski W., Kujawa J., Navia R. (2020). A Short Review on the Valorization of Green Seaweeds and Ulvan: Feedstock for Chemicals and Biomaterials. Biomolecules.

[61] Ben Amor C., Jmel M.A., Chevallier P., Mantovani D., Smaali I. (2021). Efficient Extraction of a High Molecular Weight Ulvan from Stranded *Ulva* Sp. Biomass: Application on the Active Biomembrane Synthesis. Biomass Convers. Biorefin..

[62] Zheng W., Hao Y., Wang D., Huang H., Guo F., Sun Z., Shen P., Sui K., Yuan C., Zhou Q. (2021). Preparation of Triamcinolone Acetonide-Loaded Chitosan/Fucoidan Hydrogel and Its Potential Application as an Oral Mucosa Patch. Carbohydr. Polym..

[63] Wahlström N., Nylander F., Malmhäll-Bah E., Sjövold K., Edlund U., Westman G., Albers E. (2020). Composition and Structure of Cell Wall Ulvans Recovered from Ulva Spp. along the Swedish West Coast. Carbohydr. Polym..

[64] Ganesan A.R., Shanmugam M., Bhat R. (2018). Producing Novel Edible Films from Semi Refined Carrageenan (SRC) and Ulvan Polysaccharides for Potential Food Applications. Int. J. Biol. Macromol..

[65] Aleissa M.S., Alkahtani S., Abd Eldaim M.A., Ahmed A.M., Bungău S.G., Almutairi B., Bin-Jumah M., AlKahtane A.A., Alyousif M.S., Abdel-Daim M.M. (2020). Fucoidan Ameliorates Oxidative Stress, Inflammation, DNA Damage, and Hepatorenal Injuries in Diabetic Rats Intoxicated with Aflatoxin B1. Oxid. Med. Cell. Longev..

[66] Lim S.J., Aida W.M.W., Maskat M.Y., Latip J., Badri K.H., Hassan O., Yamin B.M. (2016). Characterisation of Fucoidan Extracted from Malaysian Sargassum Binderi. Food Chem..

[67] Zhao D., Xu J., Xu X. (2018). Bioactivity of Fucoidan Extracted from Laminaria Japonica Using a Novel Procedure with High Yield. Food Chem..

[68] Shen P., Yin Z., Qu G., Wang C. (2018). Bioactive Seaweeds for Food Applications.

[69] Wang Y., Xing M., Cao Q., Ji A., Liang H., Song S. (2019). Biological Activities of Fucoidan and the Factors Mediating Its Therapeutic Effects: A Review of Recent Studies. Mar. Drugs.

[70] Wu L., Sun J., Su X., Yu Q., Yu Q., Zhang P. (2016). A Review about the Development of Fucoidan in Antitumor Activity: Progress and Challenges. Carbohydr. Polym..

[71] Luthuli S., Wu S., Cheng Y., Zheng X., Wu M., Tong H. (2019). Therapeutic Effects of Fucoidan: A Review on Recent Studies. Mar. Drugs.

[72] Chen Q., Liu M., Zhang P., Fan S., Huang J., Yu S., Zhang C., Li H. (2019). Fucoidan and Galactooligosaccharides Ameliorate High-Fat Diet–Induced Dyslipidemia in Rats by Modulating the Gut Microbiota and Bile Acid Metabolism. Nutrition.

[73] Kwak J.-Y. (2014). Fucoidan as a Marine Anticancer Agent in Preclinical Development. Mar. Drugs.

[74] Apostolova E., Lukova P., Baldzhieva A., Katsarov P., Nikolova M., Iliev I., Peychev L., Trica B., Oancea F., Delattre C. (2020). Immunomodulatory and Anti-Inflammatory Effects of Fucoidan: A Review. Polymers.

[75] Kadam S.U., Tiwari B.K., O’Donnell C.P. (2015). Extraction, Structure and Biofunctional Activities of Laminarin from Brown Algae. Int. J. Food Sci. Technol..

[76] Verdugo P., Orellana M.V., Chin W.-C., Petersen T.W., van den Eng G., Benner R., Hedges J.I. (2008). Marine Biopolymer Self-Assembly: Implications for Carbon Cycling in the Ocean. Faraday Discuss..

[77] Mitsuya D., Yamamoto M., Okai M., Inoue A., Suzuki T., Ojima T., Urano N. (2017). Continuous Saccharification of Laminarin by Immobilized Laminarinase Ulam111 Followed by Ethanol Fermentation with a Marine-Derived Yeast. Adv. Microbiol..

[78] Mišurcová L. (2011). Chemical composition of seaweeds. Handbook of Marine Macroalgae: Biotechnology and Applied Phycology.

[79] Karuppusamy S., Rajauria G., Fitzpatrick S., Lyons H., McMahon H., Curtin J., Tiwari B.K., O’Donnell C. (2022). Biological Properties and Health-Promoting Functions of Laminarin: A Comprehensive Review of Preclinical and Clinical Studies. Mar. Drugs.

[80] Manivasagan P., Oh J. (2016). Marine Polysaccharide-Based Nanomaterials as a Novel Source of Nanobiotechnological Applications. Int. J. Biol. Macromol..

[81] Praseptiangga D., Joni I.M., Tjahjono B. (2022). Advances on Biopolymers Derived from Marine and Agricultural Products for Sustainable Food Packaging Applications. Front. Sustain. Food Syst..

[82] Barikani M., Oliaei E., Seddiqi H., Honarkar H. (2014). Preparation and Application of Chitin and Its Derivatives: A Review. Iran. Polym. J..

[83] Singh R., Shitiz K., Singh A. (2017). Chitin and Chitosan: Biopolymers for Wound Management. Int. Wound J..

[84] Ali G., Sharma M., Salama E.-S., Ling Z., Li X. (2022). Applications of Chitin and Chitosan as Natural Biopolymer: Potential Sources, Pretreatments, and Degradation Pathways. Biomass Convers. Biorefin..

[85] Rebecca L.J., Susithra G., Sharmila S., Das M.P. (2013). Isolation and Screening of Chitinase Producing Serratia Marcescens from Soil. J. Chem. Pharm. Res..

[86] Claverie M., McReynolds C., Petitpas A., Thomas M., Fernandes S.C.M. (2020). Marine-Derived Polymeric Materials and Biomimetics: An Overview. Polymers.

[87] Song Z., Li G., Guan F., Liu W. (2018). Application of Chitin/Chitosan and Their Derivatives in the Papermaking Industry. Polymers.

[88] Harkin C., Mehlmer N., Woortman D.V., Brück T.B., Brück W.M. (2019). Nutritional and Additive Uses of Chitin and Chitosan in the Food Industry. Sustainable Agriculture Reviews 36: Chitin and Chitosan: Applications in Food, Agriculture, Pharmacy, Medicine and Wastewater Treatment.

[89] Roy J.C., Salaün F., Giraud S., Ferri A., Chen G., Guan J. (2017). Solubility of Chitin: Solvents, Solution Behaviors and Their Related Mechanisms. Solubility Polysacch..

[90] Merzendorfer H. (2011). The Cellular Basis of Chitin Synthesis in Fungi and Insects: Common Principles and Differences. Eur. J. Cell Biol..

[91] Roy S., Rhim J.-W. (2021). Effect of Chitosan Modified Halloysite on the Physical and Functional Properties of Pullulan/chitosan Biofilm Integrated with Rutin. Appl. Clay Sci..

[92] Joye J.I., Julian McClements D. (2016). Biopolymer-Based Delivery Systems: Challenges and Opportunities. Curr. Top. Med. Chem..

[93] Sałek K., Gutierrez T. (2016). Surface-Active Biopolymers from Marine Bacteria for Potential Biotechnological Applications. AIMS Microbiol..

[94] Monteiro L.P.G., Borges J., Rodrigues J.M.M., Mano J.F. (2023). Unveiling the Assembly of Neutral Marine Polysaccharides into Electrostatic-Driven Layer-by-Layer Bioassemblies by Chemical Functionalization. Mar. Drugs.

[95] Yuswan M.H., Jalil N.H.A., Mohamad H., Keso S., Mohamad N.A., Yusoff T.S.T.M., Ismail N.F., Manaf Y.N.A., Hashim A.M., Desa M.N.M. (2021). Hydroxyproline Determination for Initial Detection of Halal-Critical Food Ingredients (Gelatin and Collagen). Food Chem..

[96] Hashim P., Ridzwan M.M.S., Bakar J., Hashim M.D. (2015). Collagen in Food and Beverage Industries. Int. Food Res. J..

[97] Abd El-Salam M.H., El-Shibiny S. (2016). Natural biopolymers as nanocarriers for bioactive ingredients used in food industries. Encapsulations.

[98] Bhagwat P.K., Dandge P.B. (2016). Isolation, Characterization and Valorizable Applications of Fish Scale Collagen in Food and Agriculture Industries. Biocatal. Agric. Biotechnol..

[99] Vilarinho F., Sanches Silva A., Vaz M.F., Farinha J.P. (2018). Nanocellulose in Green Food Packaging. Crit. Rev. Food Sci. Nutr..

[100] Meneghetti M.C.Z., Hughes A.J., Rudd T.R., Nader H.B., Powell A.K., Yates E.A., Lima M.A. (2015). Heparan Sulfate and Heparin Interactions with Proteins. J. R. Soc. Interface.

[101] Nakamoto M.M., Assis M., de Oliveira Filho J.G., Braga A.R.C. (2023). Spirulina Application in Food Packaging: Gaps of Knowledge and Future Trends. Trends Food Sci Technol.

[102] Milovanovic I., Hayes M. (2018). Marine Gelatine from Rest Raw Materials. Appl. Sci..

[103] Rathod N.B., Bangar S.P., Šimat V., Ozogul F. (2023). Chitosan and Gelatine Biopolymer-based Active/Biodegradable Packaging for the Preservation of Fish and Fishery Products. Int. J. Food Sci. Technol..

[104] Mehetre S.S., Shankar R.K., Ameta R.K., Behere S.S. (2023). An introduction to protein-based biopolymers. Protein-Based Biopolymers.

[105] Jumaidin R. (2023). Agar based composite as a new alternative biopolymer. Composites from the Aquatic Environment.

[106] Charoenpol A., Crespy D., Schulte A., Suginta W. (2023). Marine Chitin Upcycling with Immobilized Chitinolytic Enzymes: Current State and Prospects. Green Chem..

[107] Beiras R., López-Ibáñez S. (2023). A Practical Tool for the Assessment of Polymer Biodegradability in Marine Environments Guides the Development of Truly Biodegradable Plastics. Polymers.

[108] Hamed I., Özogul F., Regenstein J.M. (2016). Industrial Applications of Crustacean By-Products (Chitin, Chitosan, and Chitooligosaccharides): A Review. Trends Food Sci. Technol..

[109] Kwon Y.-M., Moon J.H., Cho G.-C., Kim Y.U., Chang I. Xanthan Gum Biopolymer-Based Soil Treatment (Bpst) as a Construction Material to Mitigate Internal Erosion of Earthen Levee and Embankment Structures: A Field-Scale Study. https://papers.ssrn.com/sol3/papers.cfm?abstract_id=4370967.

[110] Issa A.T., Tahergorabi R. (2020). Barrier, degradation, and cytotoxicity studies for chitin-chitosan bionanocomposites. Chitin-and Chitosan-Based Biocomposites for Food Packaging Applications.

[111] Venkatesan J., Bhatnagar I., Manivasagan P., Kang K.-H., Kim S.-K. (2015). Alginate Composites for Bone Tissue Engineering: A Review. Int. J. Biol. Macromol..

[112] Williams P.A., Campbell K.T., Gharaviram H., Madrigal J.L., Silva E.A. (2017). Alginate-Chitosan Hydrogels Provide a Sustained Gradient of Sphingosine-1-Phosphate for Therapeutic Angiogenesis. Ann. Biomed. Eng..

[113] Pereira L., Gheda S.F., Ribeiro-Claro P.J.A. (2013). Analysis by Vibrational Spectroscopy of Seaweed Polysaccharides with Potential Use in Food, Pharmaceutical, and Cosmetic Industries. Int. J. Carbohydr. Chem..

[114] Khalil H.P.S.A., Saurabh C.K., Tye Y.Y., Lai T.K., Easa A.M., Rosamah E., Fazita M.R.N., Syakir M.I., Adnan A.S., Fizree H.M. (2017). Seaweed Based Sustainable Films and Composites for Food and Pharmaceutical Applications: A Review. Renew. Sustain. Energy Rev..

[115] Xiao Q., Weng H., Ni H., Hong Q., Lin K., Xiao A. (2019). Physicochemical and Gel Properties of Agar Extracted by Enzyme and Enzyme-Assisted Methods. Food Hydrocoll..

[116] Lee W.-K., Lim Y.-Y., Leow A.T.-C., Namasivayam P., Abdullah J.O., Ho C.-L. (2017). Biosynthesis of Agar in Red Seaweeds: A Review. Carbohydr. Polym..

[117] Kumar L., Ramakanth D., Akhila K., Gaikwad K.K. (2022). Edible Films and Coatings for Food Packaging Applications: A Review. Environ. Chem. Lett..

[118] Lionetto F., Esposito Corcione C. (2021). Recent Applications of Biopolymers Derived from Fish Industry Waste in Food Packaging. Polymers.

[119] Malhotra B., Keshwani A., Kharkwal H. (2015). Natural Polymer Based Cling Films for Food Packaging. Int. J. Pharm. Pharm. Sci..

[120] Bourbon A.I., Pereira R.N., Pastrana L.M., Vicente A.A., Cerqueira M.A. (2019). Protein-Based Nanostructures for Food Applications. Gels.

[121] Kaewprachu P., Osako K., Rawdkuen S. (2018). Effects of Plasticizers on the Properties of Fish Myofibrillar Protein Film. J. Food Sci. Technol..

[122] Della Malva A., Albenzio M., Santillo A., Russo D., Figliola L., Caroprese M., Marino R. (2018). Methods for Extraction of Muscle Proteins from Meat and Fish Using Denaturing and Nondenaturing Solutions. J. Food Qual.

[123] Blanco M., Vázquez J.A., Pérez-Martín R.I., Sotelo G.C. (2019). Collagen Extraction Optimization from the Skin of the Small-Spotted Catshark (*S. canicula*) by Response Surface Methodology. Mar. Drugs.

[124] León-López A., Morales-Peñaloza A., Martínez-Juárez V.M., Vargas-Torres A., Zeugolis D.I., Aguirre-Álvarez G. (2019). Hydrolyzed Collagen—Sources and Applications. Molecules.

[125] Roy S., Rhim J.-W. (2020). Preparation of Antimicrobial and Antioxidant Gelatin/curcumin Composite Films for Active Food Packaging Application. Colloids Surf. B Biointerfaces.

[126] Roy S., Ezati P., Rhim J.-W. (2022). Fabrication of Antioxidant and Antimicrobial Pullulan/gelatin Films Integrated with Grape Seed Extract and Sulfur Nanoparticles. ACS Appl. Bio Mater..

[127] Iliou K., Kikionis S., Ioannou E., Roussis V. (2022). Marine Biopolymers as Bioactive Functional Ingredients of Electrospun Nanofibrous Scaffolds for Biomedical Applications. Mar. Drugs.

[128] Nowzari F., Shábanpour B., Ojagh S.M. (2013). Comparison of Chitosan–Gelatin Composite and Bilayer Coating and Film Effect on the Quality of Refrigerated Rainbow Trout. Food Chem..

[129] Serrano-León J.S., Bergamaschi K.B., Yoshida C.M.P., Saldaña E., Selani M.M., Rios-Mera J.D., Alencar S.M., Contreras-Castillo C.J. (2018). Chitosan Active Films Containing Agro-Industrial Residue Extracts for Shelf Life Extension of Chicken Restructured Product. Food Res. Int..

[130] Jiang T., Feng L., Wang Y. (2013). Effect of Alginate/Nano-Ag Coating on Microbial and Physicochemical Characteristics of Shiitake Mushroom (Lentinus Edodes) during Cold Storage. Food Chem..

[131] Mohamed S.A.A., El-Sakhawy M., El-Sakhawy M.A.-M. (2020). Polysaccharides, Protein and Lipid-Based Natural Edible Films in Food Packaging: A Review. Carbohydr. Polym..

[132] Gamboa-Santos J., Campañone L.A. (2019). Application of Osmotic Dehydration and Microwave Drying to Strawberries Coated with Edible Films. Dry. Technol..

[133] Escamilla-García M., Rodríguez-Hernández M.J., Hernández-Hernández H.M., Delgado-Sánchez L.F., García-Almendárez B.E., Amaro-Reyes A., Regalado-González C. (2018). Effect of an Edible Coating Based on Chitosan and Oxidized Starch on Shelf Life of *Carica papaya* L., and Its Physicochemical and Antimicrobial Properties. Coatings.

[134] Rao P.S., Sharma H., Singh R., Meghawal K., Pradhan D. (2018). Chitosan and Its Application in Dairy Industry. Quality Control and Waste Utilization for Agriculture and Dairy Products.

[135] Roy S., Ezati P., Khan A., Rhim J.-W. (2023). New Opportunities and Advances in Quercetin-added Functional Packaging Films for Sustainable Packaging Applications: A Mini-review. Crit. Rev. Food Sci. Nutr..

[136] Babaremu K., Oladijo O.P., Akinlabi E. Biopolymers: A Suitable Replacement for Plastics in Product Packaging. Adv. Ind. Eng. Polym. Res..

[137] Alazaiza M.Y.D., Albahnasawi A., Eyvaz M., Al Maskari T., Nassani D.E., Abu Amr S.S., Abujazar M.S.S., Bashir M.J.K. (2023). An Overview of Green Bioprocessing of Algae-Derived Biochar and Biopolymers: Synthesis, Preparation, and Potential Applications. Energies.

[138] Basumatary I.B., Mukherjee A., Katiyar V., Kumar S. (2022). Biopolymer-Based Nanocomposite Films and Coatings: Recent Advances in Shelf-Life Improvement of Fruits and Vegetables. Crit. Rev. Food Sci. Nutr..

[139] Guerreiro A.C., Gago C.M.L., Faleiro M.L., Miguel M.G.C., Antunes M.D.C. (2016). Edible Coatings Enriched with Essential Oils for Extending the Shelf-life of ‘Bravo de Esmolfe’Fresh-cut Apples. Int. J. Food Sci. Technol..

[140] Kumar S., Boro J.C., Ray D., Mukherjee A., Dutta J. (2019). Bionanocomposite films of agar incorporated with ZnO nanoparticles as an active packaging material for shelf life extension of green grape. Heliyon.

[141] Roy S., Priyadarshi R., Rhim J.-W. (2022). Gelatin/Agar-Based Multifunctional Film Integrated with Copper-Doped Zinc Oxide Nanoparticles and Clove Essential Oil Pickering Emulsion for Enhancing the Shelf Life of Pork Meat. Food Res. Int..

[142] Gedarawatte S.T.G., Ravensdale J.T., Johns M.L., Azizi A., Al-Salami H., Dykes G.A., Coorey R. (2021). Effectiveness of Gelatine and Chitosan Spray Coating for Extending Shelf Life of Vacuum-packaged Beef. Int. J. Food Sci. Technol..

[143] Sapie S.R., Kamari A., Jumadi J. (2023). A Brief Review of Propolis as an Additive in Biopolymer Matrix Films for Food Packaging. Proceedings of the 8th International Conference on Research Implementation and Education of Mathematics and Science (ICRIEMS 2021): Transforming Science Literacy into A New Normal Digital World to Achieve Sustainable Development Goals.

[144] Wang H., Ding F., Ma L., Zhang Y. (2021). Recent Advances in Gelatine and Chitosan Complex Material for Practical Food Preservation Application. Int. J. Food Sci. Technol..

[145] Roy S., Ezati P., Biswas D., Rhim J.-W. (2022). Shikonin Functionalized Packaging Film for Monitoring the Freshness of Shrimp. Materials.

[146] Ozogul F., Elabed N., Ceylan Z., Ocak E., Ozogul Y. (2021). Nano-Technological Approaches for Plant and Marine-Based Polysaccharides for Nano-Encapsulations and Their Applications in Food Industry. Advances in Food and Nutrition Research.

[147] Zhang W., Roy S., Rhim J.-W. (2023). Copper-based Nanoparticles for Biopolymer-based Functional Films in Food Packaging Applications. Compr. Rev. Food Sci. Food Saf..

[148] Sudha P.N., Sangeetha K., Gomathi T. (2017). Introduction to marine biopolymers. Industrial Applications of Marine Biopolymers.

[149] Kim H.J., Roy S., Rhim J.-W. (2022). Gelatin/agar-based Color-indicator Film Integrated with *Clitoria ternatea* Flower Anthocyanin and Zinc Oxide Nanoparticles for Monitoring Freshness of Shrimp. Food Hydrocoll..

[150] Da Zavareze E.R., el Halal S.L.M., Marques e Silva R., Dias A.R.G., Prentice-Hernández C. (2014). Mechanical, Barrier and Morphological Properties of Biodegradable Films Based on Muscle and Waste Proteins from the W Hitemouth Croaker (M Icropogonias Furnieri). J. Food Process. Preserv..

[151] Vinayak A., Sharma S., Singh G.B. (2022). Biopolymers from industrial waste. Biopolymers: Recent Updates, Challenges and Opportunities.

[152] Qureshi D., Nayak S.K., Anis A., Ray S.S., Kim D., Nguyen T.T.H., Pal K. (2020). Introduction of Biopolymers: Food and Biomedical Applications. Biopolymer-Based Formulations.

[153] Araújo C.S., Rodrigues A.M.C., Joele M.R.S.P., Araújo E.A.F., Lourenço L.F.H. (2018). Optimizing Process Parameters to Obtain a Bioplastic Using Proteins from Fish Byproducts through the Response Surface Methodology. Food Packag. Shelf Life.

[154] Cardoso G.P., Dutra M.P., Fontes P.R., de Ramos A.L.S., de Miranda Gomide L.A., Ramos E.M. (2016). Selection of a Chitosan Gelatin-Based Edible Coating for Color Preservation of Beef in Retail Display. Meat. Sci..

[155] Alsaggaf M.S., Moussa S.H., Tayel A.A. (2017). Application of Fungal Chitosan Incorporated with Pomegranate Peel Extract as Edible Coating for Microbiological, Chemical and Sensorial Quality Enhancement of Nile Tilapia Fillets. Int. J. Biol. Macromol..

[156] Sapper M., Chiralt A. (2018). Starch-Based Coatings for Preservation of Fruits and Vegetables. Coatings.

[157] Abdelhedi O., Salem A., Nasri R., Nasri M., Jridi M. (2022). Food Applications of Bioactive Marine Gelatin Films. Curr. Opin. Food Sci..

[158] Chaudhary S., Kumar S., Kumar V., Sharma R. (2020). Chitosan Nanoemulsions as Advanced Edible Coatings for Fruits and Vegetables: Composition, Fabrication and Developments in Last Decade. Int. J. Biol. Macromol..

[159] Ju J., Xie Y., Guo Y., Cheng Y., Qian H., Yao W. (2019). Application of Edible Coating with Essential Oil in Food Preservation. Crit. Rev. Food Sci. Nutr..

[160] Ramakrishnan R., Kulandhaivelu S.V., Roy S. (2023). Alginate/carboxymethyl cellulose/starch-based Active Coating with Grapefruit Seed Extract to Extend the Shelf Life of Green Chilli. Ind. Crops Prod..

[161] Ramakrishnan R., Kulandhaivelu S.V., Roy S., Viswanathan V.P. (2023). Characterisation of Ternary Blend Film of Alginate/carboxymethyl cellulose/starch for Packaging Applications. Ind. Crops Prod..

[162] Di Donato P., Poli A., Taurisano V., Abbamondi G.R., Nicolaus B., Tommonaro G. (2016). Recent Advances in the Study of Marine Microbial Biofilm: From the Involvement of Quorum Sensing in Its Production up to Biotechnological Application of the Polysaccharide Fractions. J. Mar. Sci. Eng..

[163] Kim S.-K., Perera U., Rajapakse N., Kim S. (2016). Seafood Processing By-Products.

[164] Yousefi M., Jafari S.M. (2019). Recent Advances in Application of Different Hydrocolloids in Dairy Products to Improve Their Techno-Functional Properties. Trends Food Sci. Technol..

[165] Ebrahimzadeh S., Biswas D., Roy S., McClements D.J. (2023). Incorporation of Essential Oils in Edible Seaweed-based Films: A Comprehensive Review. Trends Food Sci. Technol..

[166] Vidanarachchi J.K., Kurukulasuriya M.S., Samaraweera A.M., Silva K. (2012). Applications of Marine Nutraceuticals in Dairy Products. Adv. Food Nutr. Res..

[167] Evdokimov I.A., Alieva L.R., Varlamov V.P., Kharitonov V.D., Butkevich T.V., Kurchenko V.P. (2015). Usage of Chitosan in Dairy Products Production. Foods Raw Mater..

[168] Vivek K. (2013). Use of Encapsulated Probiotics in Dairy Based Foods. Int. J. Food Agric. Vet. Sci..

[169] Sharma H., Mahajan G., Kaur M., Gupta R. (2023). Additives in dairy-based food. Microbes for Natural Food Additives.

[170] Zhao Y., Khalesi H., He J., Fang Y. (2023). Application of Different Hydrocolloids as Fat Replacer in Low-Fat Dairy Products: Ice Cream, Yogurt and Cheese. Food Hydrocoll..

